# Antioxidant and Anti-inflammatory Effects of Marine Phlorotannins and Bromophenols Supportive of Their Anticancer Potential

**DOI:** 10.1093/nutrit/nuae066

**Published:** 2024-06-18

**Authors:** Luis Goya, Raquel Mateos

**Affiliations:** Department of Metabolism and Nutrition, Institute of Food Science, Technology, and Nutrition (ICTAN-CSIC), Spanish National Research Council (CSIC), 28040 Madrid, Spain; Department of Metabolism and Nutrition, Institute of Food Science, Technology, and Nutrition (ICTAN-CSIC), Spanish National Research Council (CSIC), 28040 Madrid, Spain

**Keywords:** phlorotannins, bromophenols, cancer, marine organisms, antioxidant activity, anti-inflammatory activity

## Abstract

Following the goal of optimizing nutrition, the food industry has been continuously working on food reformulation, nutritional patterns, functional foods development, and the general promotion of a healthy lifestyle. To this end, the scientific community has been increasingly investigating natural compounds that could prevent or treat chronic diseases. Phlorotannins and bromophenols are phenolic compounds particularly present in marine organisms. There is extensive evidence that shows their potential in the prevention of noncommunicable diseases, including cancer, the second cause of mortality worldwide. Numerous studies have demonstrated the anticarcinogenic activity of polyphenolic algae compounds both in cell culture and experimental animal models. Although recent reviews are also available, the present update focuses on the most recent findings related to the antioxidant/anti-inflammatory effect of seaweed phenolics, as well as their regulatory capacity for new molecular targets. Additionally, the review addresses and discusses the close link between inflammation and oxidative stress, along with their relationship with tumor onset and progression, including the most recent findings supporting this correlation. Although clinical studies are still needed to support this evidence, phlorotannins and bromophenols constitute an emerging bioactive group with high potential as chemopreventive agents and/or potential adjuvants for existing cancer therapies.

## INTRODUCTION

Cancer is the second leading cause of death worldwide, accounting for nearly 10 million deaths in 2020, or nearly 1 in 6 deaths. The most common cancers are breast, lung, colon and rectum, and prostate cancers.^1^ Cancer arises from the transformation of normal cells into tumor cells in a multistage process that generally progresses from a precancerous lesion to a malignant tumor. The complex biological processes of cancer initiation and progression involve multiple steps, resulting in heterogeneity of tumor tissue and the surrounding microenvironment.^2^ Standard cancer treatment typically entails the use of cytotoxic or selectively targeted anticancer agents in combination with surgical interventions and radiation therapy. Another promising approach that has been implemented during recent years involves nutritional prevention and/or supplementation as a coadjutant strategy; one of the aims of optimal nutrition, the ideal intake of nutrients for an individual in order to achieve optimal health, is defined as the most efficient functioning of the human organism body and mind.^3^ The research has been mainly focused on phytochemicals, plant-derived nutritive and nonnutritive compounds with health-promoting activities, such as antioxidant, anti-inflammatory, and anticancer activities.^3^^,^^4^

Marine organisms are a rich source of bioactive compounds less explored than terrestrial sources, although with promising applications in the prevention of cancer.^3^^,^^4^ Thus, the National Cancer Institute estimated that approximately 1% of marine natural products showed anti-tumor cytotoxicity properties compared with only 0.01% of their terrestrial counterparts.^5^ Therefore, identifying new marine natural products is emergent research to improve existing therapies and develop novel cures. Among marine metabolites with biological properties, phenolic compounds constitute the largest family of secondary metabolites. Although they are ubiquitous throughout the plant kingdom, bromophenols and phlorotannins are considered more specific to marine sources in comparison to phenolic acids or flavonoids.^4^ The production of phenolic compounds in marine organisms occurs naturally and is linked to external factors, particularly environmental stressors. These stressors may include desiccation, salinity, ultraviolet (UV) radiation, nutrient availability, and temperature.^6–8^ Marine polyphenolic compounds have been demonstrated to have potent anticarcinogenic activity and have been exhaustively reviewed by Erpel et al,^3^ Mateos et al,^4^ and very recently by Besednova et al^9^ and Matulja et al^10^; however, some recent findings related to the antioxidant/anti-inflammatory effect of these seaweed phenolics, as well as their regulatory capacity on new molecular targets, could be added. Even though all of the above studies have reported on the crucial role of inflammation in the neoplastic process, especially in the study from Besednova and colleagues,^9^ the intimate relation between inflammation and oxidative stress and of these 2 processes with tumor onset and progression deserves further attention, which will be discussed in-depth in this review.

## SEARCH STRATEGY

Using the PubMed and Web of Science databases, an extensive literature search of original articles published in recent years was conducted. The search utilized the following terms: “phlorotannins”, “bromophenols”, “cancer”, “marine organisms”, “antioxidant activity”, and “anti-inflammatory activity”. No additional restrictions were applied beyond these selected search terms. Initially, all search results underwent screening for potential inclusion based on their publication titles and abstracts. Articles deemed irrelevant based on their headings and subheadings were omitted. Eligible publications for inclusion in the review underwent careful assessment through full-text review and discussion when necessary. Moreover, the references of the selected papers were searched for other relevant manuscripts.

## PHENOLIC CLASSES

**Figure 1.**
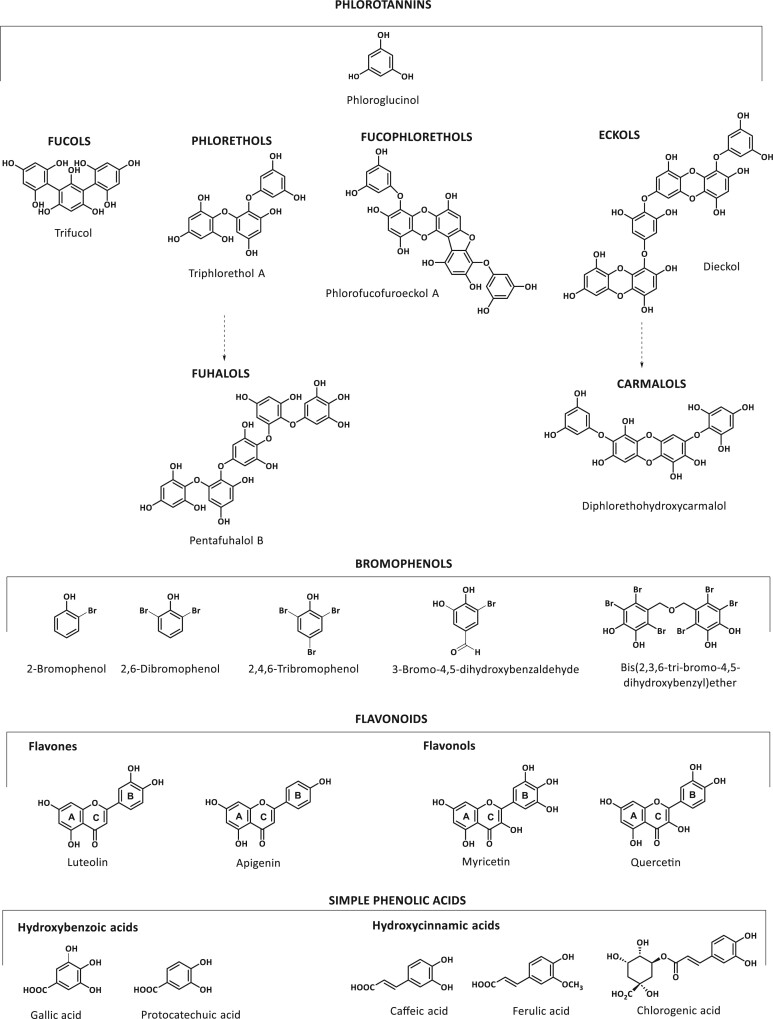
Chemical Structures of the Main Marine Polyphenols: Phlorotannins, Bromophenols, Flavonoids, and Simple Phenolic Compounds

Phenolic molecules are characterized by the presence of an aromatic ring with 1 or more hydroxyl groups and broad structural variability from simple molecules, such as phenolic acids to more complex polyphenolic polymers.^11–14^ Complex polymers of phloroglucinol (1,3,5-trihydroxybenzene), known as phlorotannins, are primarily found in brown algae, predominantly Laminariaceae, Lessoniaceae, and Fucaceae families. This group shows a varied chemical structure according to the different way that phloroglucinol units are linked and the variable degree of polymerization (126 KDa–650 kDa).^15^ The inter-monomeric linkages determine the different groups of phlorotannins as follows: fucols possess only aryl–aryl linkages, phlorethols with aryl–ether linkages, and fucophlorethols possess aryl–aryl and aryl–ether units and eckols, which contain a 3-ring structure with a dibenzodioxin moiety substituted by phloroglucinol at C-4. Within the subclass of phlorethols, there are also the fuhalols, with aryl–ether linkages and an additional hydroxyl group. Likewise, eckols with additional hydroxyl group are called carmalols (Figure 1).^4^

Bromophenols are the other specific family of phenolic compounds, particularly prevalent in marine sources. They have been identified in several macroalgae (red, green, and brown). Bromophenols are formed after the action of vanadium peroxidases, in charge of catalyzing the bromination of phenolic rings in algae.^16^ The presence of these compounds in these organisms is demonstrated due to their aromatic properties since they are responsible for algae flavor attributed to 2-bromophenol, 2,6-bromophenol, and 2,4,6-tribromophenol (Figure 1).^16^

Flavonoids are low-molecular-weight molecules that are not exclusive to terrestrial plants, as they are also present in marine sources. Flavonoids are usually found in algae in glycosylated forms. This category includes (in order of abundance in marine sources) flavones (luteolin, apigenin, chrysin, and baicalein), flavonols (myricetin, kaempherol, and quercetin), flavanones, anthocyanins, and isoflavones. Catechins have also been detected, particularly epicatechin and epigallocatechin. Most of the flavonoids isolated from marine sources come from seagrasses and halophytes and have been investigated for their pharmacological activities (Figure 1).^17^

The simplest phenolic acids are also characterized in marine or freshwater organisms. Gallic acid, protocatechuic acid, chlorogenic acid, caffeic acid, gentisic acid, and vanillic acid have been found in marine species (Figure 1).^3^^,^^4^^,^^17^^,^^18^

## BIOCHEMICAL AND MOLECULAR MECHANISMS OF ACTION REGULATED BY PHLOROTANNINS AND BROMOPHENOLS

Anticancer activities of marine-derived phenolic compounds such as phlorotannins and bromophenols have been extensively reviewed.^3^^,^^4^^,^^9^^,^^10^ However, antioxidant/anti-inflammatory effects of these seaweed phenolics as well as their regulatory capacity on new molecular targets could be reviewed to complete the knowledge on the bioactivity of these compounds. Additionally, the intimate relation between inflammation and oxidative stress and of these 2 processes with tumor onset and progression deserves further attention.

In order to present the most recent findings regarding information displayed in the present manuscript, a thorough search in PubMed (MEDLINE database) introducing terms such as phlorotannins, phloroglucinol, bromophenols, algae polyphenols, and seaweed phenolics alone and together with anticancer, antiproliferative, antitumor, and derivatives was carried out for the last 5 years.

### Recent advances on effects related to oxidative stress and inflammation

#### Oxidative stress

Continuous exposure to pollution carcinogens, UVB radiation, tobacco smoke, unhealthy foodstuff, and several viral infections has essentially been the cause of increased cancer prevalence.^19^ Disproportionate reactive oxygen species (ROS) production by these xenobiotics is unmanageable by cellular antioxidant defenses and results in oxidative stress. The overproduction of oxygen- and nitrogen-containing metabolites as free radicals, along with inflammatory and immune response signaling, plays an essential role in the mechanism of cell pathogenesis.^9^ ROS and reactive nitrogen species (RNS), generated from cell metabolism, play a significant role in the activation of inflammatory pathways mediated by protein kinases, transcription factors, and enhanced genomic expression of proinflammatory factors.^20^^,^^21^ Increased and/or maintained ROS production is intimately related to the development of most chronic inflammatory diseases. Thus, since secretion of proinflammatory cytokines is directly associated with the concentration of ROS,^22^ oxidative stress usually accompanies the onset and development of the inflammatory process.^23^ These facts substantiate the common pathophysiological mechanisms between inflammation and oxidative stress.^24^^,^^25^ The outcome of the combined effect of both processes implies modification of the assembly of cell proteins and lipids, alteration of the mechanisms of cell transport and proliferation, and triggering of Toll-like receptor (TLR) and immune system cells, with enhanced production of proinflammatory cytokines and cell death by apoptosis.^26^

In cells, the levels of ROS are controlled by the cell antioxidant defense system, which includes enzymatic (glutathione peroxidase [GPx], glutathione reductase [GR], glutathione-*S*-transferase [GST], catalase [CAT], and others) and nonenzymatic (classically reduced glutathione [GSH]) components.^21^^,^^27^ However, this endogenous defense system can be helped by exogenous natural antioxidants, such as plant polyphenols, which reinforce and reinstate optimal balance by neutralizing ROS and enhancing defenses.^28^^,^^29^ Seaweed’s polyphenolic compounds have strong antioxidant activity,^27^^,^^30–35^ which is closely related to phenolic rings, which function as electron traps for the quenching of peroxide and superoxide anions and hydroxyl radicals.^36^^,^^37^

Although pioneering research reported that antioxidant capacity of polyphenols was mainly exercised through the straight deactivation of free radicals and chelating metals Fe^2+^ and Cu^+^,^38^^,^^39^ more recent studies have also included other cellular and molecular mechanisms such as enhancement of mitochondrial biogenesis through the activation of NAD-dependent protein deacetylase sirtuin-1 (SIRT1) and nuclear factor–erythroid 2 related factor 2 (Nrf2) signaling pathways and regulation of protein kinases, such as mitogen-activated protein kinases (MAPK).^39^^,^^40^ Nrf2 is a key transcription factor that regulates antioxidant responses by binding to the antioxidant response element (ARE) in the promoter region of specific antioxidant genes and promoting their transcription.^40^ To trigger this transcriptional effect, binding of Nrf2 to Kelch-like–ECH-associated protein 1 (KEAP1) in the cytoplasm must be disrupted so that Nrf2 may translocate to the nucleus, an event that may be achieved by a condition of oxidative stress and several bioactive compounds.^40^ Antioxidant defense and drug-metabolizing enzymes, such as GST, NAD(P)H–quinone oxidoreductase-1 (NQO1), heme oxygenase-1 (HO-1), UDP-glucuronosyl transferase (UGT), as well as multidrug-resistance–associated proteins, are potential targets of this crucial transcriptional mechanism.^40^ In this sense, activation of the antioxidant system by seaweed antioxidants that prevent the production of ROS and neutralize free radicals can be a fundamental constituent of the nutritional and/or pharmacological strategy for preventing and treating inflammatory diseases (Table 1).^21^^,^^28^

**Table 1.** Effect of Marine Phenolics in the Prevention of Cancer Focused on Effects Related to Oxidative Stress and Inflammation

Compounds/marine source	Test model	Outcome	Ref
Eckol from brown seaweed *Ecklonia cava* (Phaeophyceae)	In vitro: lung fibroblast V79-4 cells	Upregulated Nrf2 expression–mediated HO-1 induction via ERK and PI3K/Akt signaling	^44^
Eckol from brown algae *Ecklonia stolonifera*	In vitro: hepatocellular carcinoma HepG2 cells	Enhanced HO-1 expression mediated Nrf2 activation via JNK and PI3K/Akt signaling	^51^
Methanolic extract from brown seaweeds *Eisenia bicyclis* and *Sargassum fusiforme* (formerly *Hizikia fusiformis*) (Phaeophyceae)	In vitro: T-butyl-hydroperoxide–stimulated murine macrophage RAW 264.7 cells	Prevented the overgeneration of ROS induced by t-BOOH	^52^
Eckol	In vivo: CCl_4_-induced acute liver injury in mice	-Reduced MDA and enhanced SOD and GPx activities and GSH content -Inhibited TNF-α, IL-1, and IL-6 elevation	^53^
3-Bromo-4,5-dihydroxybenzaldehyde isolated from marine red algae	In vitro: oxygen and glucose deprivation in cardiomyocytes In vivo: myocardial ischemia and reperfusion injury induced by coronary artery ligation in rats.	-Decreased apoptosis and cleavage of caspase-3 -Decreased ROS production and lipid peroxidation -Decreased mitochondrial dysfunction -Enhanced IDH2, GPx, and SOD2 activities of the mitochondria	^54^
Eckol isolated from *Ecklonia cava* (*Phaeophyceae*)	In vitro: PM_2.5_-stimulated skin HaCaT cells	-Enhanced ROS generation -Inhibited apoptosis by inhibiting MAPK signaling pathway	^55^
Phloroglucinol	In vitro: skin HaCaT cells stimulated with H_2_O_2_	-Reverted ROS production DNA damage and apoptosis -Enhanced expression of HO-1 by the activation of Nrf2	^56^
Diphlorethohydroxycarmalol from brown algae *Ishige okamurae Yendo*	In vitro: retinal pigment epithelial ARPE19 cells stimulated with H_2_O_2_	-Reverted ROS production DNA damage and apoptosis -Reduced Bax/Bcl-2 ratio and caspase-9 and -3 activation -Inhibited ADP-ribose polymerase cleavage related to cytochrome c release	^57^
Phlorotannins from brown macroalgae *Fucus vesiculosus (Phaeophyceae)*	In vitro: LPS-stimulated macrophage RAW 264.7 cells	-Prevented NF-κB activation by inhibiting phosphorylation of upstream protein kinases -Blocked the inflammatory cascade at transcriptional level	^59^
Phlorotannin-rich extract from brown seaweed *Ecklonia cava*	In vitro: H_2_O_2_-stimulated pheochromocytoma (PC-12) and human neuroblastoma (SH-SY5Y)	-Reduced intracellular oxidative stress -Inhibited acetylcholinesterase and butyrylcholinesterase in a dose-dependent manner	^61^
Phlorotannins from brown algae *Ecklonia cava*	In vitro: irradiated mouse skin	-Decreased epidermal and dermal thickness -Enhanced Nrf2/HO-1 pathway and attenuated radiation-induced NF-κB and inflammasome activation	^62^
The bromophenol bis(2,3,6-tribromo-4,5-dihydroxybenzyl)ether (BTDE) isolated from marine red algae (particularly genera *Rhodomela confervoides and Symphyocladia latiuscula*)	In vitro: H_2_O_2_-stimulated human lung cancer A549 cells	-Decreased ROS, MDA, and GSSG/GSH ratio and increased SOD activity -Inhibited KEAP1 expression and increased Nrf2 expression and its downstream proteins TrXR1, HO-1, and NQO1	^63^
Extract from brown seaweed *Ishige okamurae* rich in diphlorethohydroxycarmalol and ishophloroglucin A	In vitro: AGE-products, induced oxidative stress in mouse glomerular mesangial cells	-Suppressed ROS production, MGO accumulation, and apoptotic cell death -Modulated proteins expression involved in the receptor for AGEs and Nrf2/ARE signaling pathways	^64^
Phlorotannin-rich extract from *Ecklonia cava* and dieckol	In vitro: neuronal pheochromocytoma PC-12 cells	-Increased cell viability -Decreased intracellular oxidative stress -Reduced pro-apoptotic proteins Bax and caspase-3 production -Decreased early and late apoptosis in PC-12 cells	^65^
Brown seaweed *Ecklonia maxima* extract containing dieckol and eckmaxol	In vitro: a) alpha-melanocyte–stimulating hormone-stimulated B16F10 cells; b) UVB-stimulated HaCaT cells; c) UVB-irradiated HDF cells	-Inhibited mushroom tyrosinase and melanogenesis in B16F10 cells -Suppressed UVB-induced HaCaT cell death consistent with inhibition of apoptosis and reduction of ROS -Inhibited collagen degradation and matrix metalloproteinases expression in UVB-irradiated HDF cells	^67^
Dioxinodehydroeckol from brown algae *Ecklonia cava*	In vitro: MCF-7 human breast cancer cells	-Increased apoptosis -Increased caspases expression and pro-apoptotic proteins p53 and Bax and reduced anti-apoptotic protein Bcl-2 expression -Reduced inflammatory transcription factor NF-κB	^76^
Brown algae *Ecklonia cava* ethanolic extracts	In vitro: lipopolysaccharide (LPS)-stimulated murine BV2 microglia	-Inhibited LPS-induced NO and PGE2 production as well as iNOS and COX-2 -Reduced NF-κB and DNA-binding in cells and MAPK activation -Suppressed proinflammatory cytokines	^77^
Brown algae *Ecklonia cava* extract containing dieckol	In vitro: LPS-stimulated murine BV2 microglia	Suppressed receptor activator of NF-κB ligand (RANKL)-induced osteoclastogenesis via MAPK/NF-κB pathway inhibition and HO-1 induction	^82^
Fucofuroeckol-A from brown algae *Eisenia bicyclis*	In vitro: LPS-induced RAW 264.7 mouse macrophages	-Suppressed NO and PGE2 production and iNO and COX-2 expression -Reduced proinflammatory cytokine production (IL-6 and TNF-α and monocyte chemoattractant protein-1) -Reduced NF-κB and MAPL activation.	^86^
An ethanolic extract from brown algae *Sargassum horneri* (Phaeophyceae)	In vitro: LPS-stimulated RAW 264.7 macrophages	-Inhibited NF-κB translocation and binding to DNA -Decreased TNF-α, IL-1, IL-6, iNOS, COX-2, NO, and PGE2	^90^
A crude ethanolic extract from red algae *Eucheuma denticulatum* (Rhodophyta)	In vitro: interferon-gamma (IFN-γ)/LPS–stimulated murine macrophage cell line (RAW 264.7)	Inhibited NO, TNF-α, IL-1β, IL-6, and MCP-1	^91^
Pyrogallol-phloroglucinol-6,6-bieckol from *Ecklonia cava*	In vivo: diet-induced obesity and leptin-deficient mice	Showed anti-inflammatory effects through regulation of TLR4 expression, ER stress, NF-κB expression, and phosphorylated STAT3	^92^
Eckol	In vitro: Reg3A-induced proliferation of human SW1990 pancreatic cancer cells	Downregulated JAK2, STAT3, NF-κB signaling pathways, and cyclin D1 protein	^93^
Aqueous extracts from *Undaria pinnatifida* (Phaeophyceae)*, Gracilariopsis longissima* (formerly *Gracilaria verrucosa*) *(*Rhodophyta) and *Codium fragile* (Chlorophyta)	In vitro: LPS-stimulated C2C12 mouse skeletal muscle cells	Decreased TNF-α levels in LPS-treated myotubes	^94^
Eckmaxol isolated from *Ecklonia maxima*	In vitro: particulate-matter–induced inflammation in MH-S lung macrophages	-Decreased COX-2 and iNOS expression -Downregulated NO, PGE2, and proinflammatory cytokines (IL-1β, IL-6 and TNF-α) -Suppressed TLR activation -Downregulated signaling of NF-κB	^95^

Abbreviations: AGE, advanced glycation end product; COX-2, cyclooxygenase-2; ER, endoplasmic reticulum; ERK, extracellular signal-regulated kinase; GPx, glutathione peroxidase; GSH, reduced glutathione; GSSG, oxidized glutathione; HO-1, heme oxygenase-1; IL, interleukin; iNOS, inducible nitric oxide synthase; JAK2, Janus kinase-2; JNK, c-Jun N-terminal kinases; KEAP1, Kelch-like–ECH-associated protein 1; MAPK, mitogen-activated protein kinases; MDA, malondialdehyde; MGO, methylglyoxal; NF-κB, nuclear transcription factor–kappaB; NO, nitric oxide; Nrf2, nuclear factor–erythroid 2 related factor 2; PGE2, prostaglandin E2; PI3K/Akt, phosphoinositide 3-kinase/protein kinase B; Ref, reference; ROS, reactive oxygen species; SOD, superoxide dismutase; STAT3, signal transducer and activator of transcription 3; t-BOOH, tert-butyl-hydroperoxide; TLR, Toll-like receptor; TNF-α, tumor necrosis factor alpha; UVB, ultraviolet B.

From the pioneering studies of more than 1 decade ago,^41–49^ to the most recent reviews,^9^^,^^10^^,^^33^^,^^50^ the antioxidant effect of seaweed phlorotannins, especially fluoroglucinol, and bromophenols on cellular markers of redox status and their antioxidative stress potential has been well documented.

Although the review will focus on the findings from the last 5 years, it is worth noting that data from 2010 indicated that phlorotannins enhance the expression of Nrf2 and HO-1 proteins, which protect from oxidative stress and inflammation. In fact, Kim et al^44^ showed that eckol, from brown seaweed *Ecklonia cava* (*Phaeophyceae*), induced HO-1 mRNA expression and protein levels activating the Nfr2/HO-1 signaling pathway to protect lung fibroblast cells from oxidative stress–induced DNA damage and apoptosis. A few years later, enhancement of HO-1 expression in a human hepatocellular carcinoma cell line (HepG2 cells) by eckol from brown algae *Ecklonia stolonifera*, mediated by activation of the Nrf2 signaling pathway through c-Jun N-terminal kinases (JNK) and phosphoinositide 3-kinase/protein kinase B (PI3K/Akt), supported the possibility of using this phlorotannin as a natural antioxidant and cytoprotector.^51^ Similarly, Han and colleagues^52^ observed that complete methanolic extracts from brown seaweeds *Eisenia bicyclis* and *Sargassum fusiforme* (formerly *Hizikia fusiformis*; *Phaeophyceae)* prevented overgeneration of ROS induced by tert-butylhydroperoxide (*t*-BOOH) in murine macrophage (RAW 264.7) cells.^52^

Focusing on the last 5 years, in 2018, eckol was reported to reduce malondialdehyde (MDA) concentration, and to increase superoxide dismutase (SOD) and GPx activities and GSH content in the liver of CCl_4_-treated mice. Moreover, eckol suppressed the CCl_4_-induced augmentation of the proinflammatory cytokines tumor necrosis factor (TNF)-alpha, interleukin (IL)-1, and IL-6 to significantly ameliorate CCl_4_-induced acute liver injury.^53^ Still, in 2018, 3-bromo-4,5-dihydroxybenzaldehyde (BDB), a natural bromophenol isolated from marine red algae, displayed antioxidant and free radical scavenging activities. BDB significantly decreased chemically induced oxidative stress, as evidenced by a significant decrease in ROS and lipid peroxidation, as well as mitochondrial disruption, determined by mitochondrial reporter gene, cytochrome c release, and adenosine triphosphate (ATP) synthesis. In addition, BDB enhanced the specific activity of mitochondrial antioxidant enzymes GPx, SOD2, and isocitrate dehydrogenase 2 (IDH2).^54^

A year later, Zhen and colleagues^55^ reported that eckol protects skin HaCaT cells from particulate matter–induced apoptosis via inhibiting ROS generation. In the same year and cell culture model, the protective effect of phloroglucinol on oxidative stress–induced DNA damage and apoptosis through activation of the Nrf2/HO-1 signaling pathway in HaCaT human keratinocytes was shown.^56^ Also in the same year, Park and coworkers^57^ showed that diphlorethohydroxycarmalol, a major phlorotannin of the brown algae *Ishige okamurae Yendo*, protected cultured ARPE19 retinal pigment epithelial cells from H_2_O_2_-induced apoptosis. This chemo-protection was mediated by a reduced Bcl-2–associated X protein/B-cell lymphoma protein 2 (Bax/Bcl-2) ratio, a decrease in caspase-9 and -3, and the inhibition of poly(adenosine diphosphate [ADP]-ribose) polymerase cleavage, which was associated with the blockage of cytochrome c release to the cytoplasm. This antioxidative stress potential is a consequence of the high polyphenolic composition reported for complete aqueous extracts from seaweeds such as *Ulva lactuca* (Chlorophyta), *Ecklonia maxima* (Phaeophyceae), *Gelidium pristoides*, and *Gracilaria gracilis* (Rhodophyta).^58^

Another year later, extracts rich in phlorotannins from brown macroalgae *Fucus vesiculosus* (Phaeophyceae) prevented the activation of nuclear factor–kappa B (NF-κB) and blocked the inflammatory cascade at the transcriptional level in LPS-stimulated macrophage RAW 264.7 cells.^59^ In the same year, enriched phlorotannin fraction from the brown seaweed *Fucus spiralis* reduced ROS production induced by H_2_O_2_ and by UVB.^60^ Similarly, phlorotannins from the edible brown seaweed *E cava* reduced intracellular oxidative stress induced by H_2_O_2_, both in pheochromocytoma (PC-12) and human neuroblastoma (SH-SY5Y) cells.^61^ Also studying phlorotannins from brown algae *E cava*, Yang and colleagues^62^ showed that these compounds decreased epidermal and dermal thickness, supporting the alleviation of acute inflammation in irradiated mouse skin. Western blotting showed that ethanolic extracts rich in phlorotannins enhanced the Nrf2/HO-1 pathway and attenuated radiation-induced NF-κB and inflammasome activation.

Furthermore, in 2021, a report from Dong and colleagues^63^ showed that the natural bromophenol *bis*(2,3,6-tribromo-4,5-dihydroxybenzyl) ether (BTDE), isolated from marine red algae (particularly genera *Rhodomela confervoides* and *Symphyocladia latiuscula*), decreased H_2_O_2_-induced ROS production, reduced MDA levels, diminished the oxidized glutathione (GSSG)/GSH proportion, and augmented the SOD activity in human lung cancer A549 cells. In the same study, BTDE repressed KEAP1 expression and stimulated that of Nrf2 and its downstream proteins TrXR1, HO-1, and NQO1. In the same year, an extract from brown seaweed *I okamurae* rich in bioactive phlorotannins such as diphlorethohydroxycarmalol and ishophloroglucin A showed protective effects against advanced glycation end product (AGE)–induced oxidative stress in mouse glomerular mesangial cells.^64^ Thus, the extract successfully suppressed intracellular ROS production, buildup of intracellular methylglyoxal (MGO), and apoptotic cell death provoked by oxidative stress induced by methylglyoxal. The protective mechanism was mediated by regulating the expression of proteins involved in the receptor for AGEs and Nrf2/ARE signaling pathways.^64^ Similarly, an *E cava* phlorotannin-rich extract and the phlorotannin dieckol showed a significant antioxidant capacity on neuronal pheochromocytoma PC-12 cells.^65^ Additionally, Rajan et al^66^ showed the activation of the Nfr2-MAPK signaling pathway in liver cells by dieckol to reduce liver cancer in animal models.

Finally, as recently as in 2022, a phlorotannin-enriched extract from brown seaweed *E maxima* mainly containing dieckol and eckmaxol, significantly suppressed UVB-induced HaCaT cell death through scavenging of over-generated intracellular ROS in a concentration-dependent manner.^67^ Thus, modulation of the Nfr2/HO-1 signaling pathway by seaweed phlorotannins may be a promising approach for preventing and treating inflammatory diseases (Table 1).

#### Inflammation

Inflammation is a multilayered biological response of body tissues to harmful insults, and its regulation involves chemokines, cytokines, TLRs, and transcription factors; most of them are responsive to natural antioxidants, especially polyphenols. TLR signaling regulates cell-mediated immunity and facilitates the inflammatory process through speeding the cellular secretion of molecules involved in pathophysiological processes, such as cytokines and chemokines. Thus, TLR signaling is crucial in the host's defense against infectious and autoimmune diseases and cancer.^68^ Within the family of TLRs, TLR2 and TLR4 (TLR2/4) activate NF-κB, which is a major inflammation regulator involved in the inducible expression of proinflammatory mediators such as inducible nitric oxide synthase (iNOS) and cyclooxygenase-2 (COX-2) and their reaction products nitric oxide (NO) and prostaglandin E2 (PGE2), as well as TNF-α, and IL-1β.^68^ Finally, sirtuins (histone deacetylases) play a main role in the pathogenesis of inflammatory diseases by controlling the expression of NF-κB. Increased expression of sirtuins may have an anti-inflammatory effect, since upregulation of Sirtuin 1 expression suppresses NF-κB transcriptional activity.^69^

Chronic inflammation is considered as a critical step in the onset and development of several types of cancer. Chronic inflammation acts as a trigger during cancer progression in the malignant transformation of cells.^70^^,^^71^ The hallmark for the development of chronic inflammation is a rise in the activity of the proinflammatory enzymes iNOS and COX-2, which generates a microenvironment that facilitates the onset of pre-neoplastic lesions.^72^ Actually, explicit inhibition of these 2 proinflammatory enzymes showed protective effects against tumor growth in several animal models, confirming them as essential targets for tumorigenesis.^72^ It has been reported that a proinflammatory cell microenvironment may stimulate mutation rates and/or favor the proliferation of mutated cells.^70^^,^^72^^,^^73^ Inflammatory cells secrete cytokines such as TNF-α to stimulate ROS accumulation in adjacent epithelial cells.^71^^,^^74^ Likewise, Grivennikov and Karin^75^ reported that the redox-sensitive NF-κB that activates iNOS and COX-2 expression is constitutively increased in neoplastic cells and may represent a risk factor for cancer development. Finally, proinflammatory cytokines produced by immune cells complete the inflammatory microenvironment. Consequently, stimulated inflammatory cells are sources of ROS and inflammatory cytokines that may provoke DNA damage and genomic instability. The accepted role of TNF-α and IL-6 as central regulators of inflammation and tumorigenesis makes them promising goals for complementary treatment in cancer.^75^ Therefore, the utilization of bioactive natural compounds that inhibit or reduce inflammation seems to be a valuable approach to delay the onset and advancement of several types of cancer.

Pioneering studies on seaweed phlorotannins and bromophenols on inflammatory processes showed that dioxinodehydroeckol from brown algae *E cava* increased apoptosis and reduced the inflammatory transcription factor NF-κB in breast cancer cells.^76^ Still, in 2009, the addition of an ethanolic extract of *E cava* rich in dieckol inhibited NF-κB translocation and regulated biosynthesis of proinflammatory cytokines in LPS-stimulated BV2 microglial cells.^77^ The same dieckol, obtained from the brown alga *E cava*, has been recurrently reported to provoke AKT/IkB-mediated NF-κB inactivation in different human cell lines.^78–85^ Another phlorotannin from the brown algae *E bicyclis*, Fucofuroeckol A, reduced the LPS-induced overproduction of NO and PGE2 and decreased the mRNA expression and protein concentration of iNOS and COX-2, and the production of proinflammatory cytokines (TNF-α, IL-6), monocyte chemoattractant protein-1, and activation of NF-κB in a culture of mouse RAW 264.7 macrophages.^86–89^

The addition of an ethanolic extract from brown algae *Sargassum horneri* (Phaeophyceae) on LPS-stimulated RAW 264.7 macrophages inhibited NF-κB translocation to the nucleus and binding to DNA to produce a dose-dependent decrease in TNF-α, IL-1, IL-6, iNOS, COX-2, NO, and PGE2.^90^ In a similar cell culture model, interferon-γ/LPS–stimulated RAW 264.7 cells, a crude ethanolic extract from red algae *E*ucheuma *denticulatum* (Rhodophyta) evoked inhibition of NO, TNF-α, IL-1β, IL-6, and monocyte chemoattractant protein-1 (MCP-1) in dose-dependent manner.^91^ Some years later, in 2019, and in the same cell culture, RAW 264.7 macrophages, extracts rich in phlorotannins from the brown alga *F vesiculosus* evoked the inhibition of the transcriptional activity of NF-κB by inhibiting phosphorylation of upstream protein kinases.^59^ In the same year, but in an in vivo approach, pyrogallol-phloroglucinol-6,6-bieckol, another compound from *E cava*, showed anti-inflammatory effects through regulation of TLR4 expression, endoplasmic reticulum (ER) stress, NF-κB expression and phosphorylated signal transducer and activator of transcription 3 (STAT3) in obese mice.^92^ Finally, eckol protected against pancreatic cancer progression by downregulation of Janus kinase-2 (JAK2), STAT3, and NF-κB signaling pathways and cyclin D1 protein.^93^

Most of the previous results have been well reviewed by Besednova and coworkers.^9^ More recently, aqueous extracts from *Undaria pinnatifida* (Phaeophyceae), *Gracilariopsis longissima* (formerly *Gracilaria verrucosa*) (Rhodophyta), and *Codium fragile* (Chlorophyta) decreased TNF-α levels in LPS-treated myotubes.^94^ In 2022, eckmaxol, a phlorotannin isolated from *E maxima* showed a protective effect against inflammation induced by particulate matter in MH-S lung macrophage cells. Eckmaxol diminished the expression of COX-2 and iNOS and downregulated NO, PGE-2, and proinflammatory cytokines (IL-1β, IL-6, and TNF-α). Furthermore, eckmaxol suppressed the activation of TLRs and downstream signaling of NF-κB.^95^ In the same year, a phlorotannin-enriched extract of brown seaweed *E maxima* containing mainly dieckol and eckmaxol exhibited strong anti-inflammatory, anti-melanogenesis, and photoprotective activities both in human epidermal HaCaT keratinocytes and human dermal fibroblasts (Table 1).^67^

## RECENT ADVANCES ON EFFECTS RELATED TO MOLECULAR PATHWAYS

### Apoptosis

Apoptosis is a fundamental mechanism of cell death regulating proliferation and tissue growth in multicellular organisms. Through apoptosis, redundant and potentially harmful cells are eliminated. Thus, the stimulation of apoptosis represents one of the primary defenses against cancer.^96^ Indeed, for years, treatment of several cancers has been addressed by induction of apoptosis through irradiation and the drug cisplatin, although with undesirable collateral effects.^97^^,^^98^ There are 2 main signaling pathways to activate caspases: the intrinsic/mitochondrial pathway, triggered by different stimuli, such as ROS and the pro-apoptotic members of Bcl-2 proteins, and the extrinsic/death receptor pathway, activated by the binding of death ligands to particular cell surface receptors (eg, fatty acid synthase [FAS]). Both stimuli increase mitochondria permeability and release of cytochrome C into the cytosol, which activates caspases.^99^ Thus, the main players of the apoptotic process include antiapoptotic Bcl-2 and proapoptotic proteins B-cell lymphoma protein 2 (Bcl-2)–associated X (Bax), as well as cysteine proteases (caspases), whose activation guarantees that the cellular components are degraded in a regulated manner, provoking cell death with minimal effect on surrounding tissues.^99^ Caspases, proteolytic enzymes that disrupt cellular structures to provoke cell death, have been broadly classified by their known roles in apoptosis (caspase-3, -6, -7, -8, and -9 in mammals) and in inflammation (caspase-1, -4, -5, -12) in humans, whereas the functions of caspase-2, -10, and -14 are less easily categorized. Caspases involved in apoptosis have also been categorized by their mechanism of action and are either initiator caspases (caspase-8 and -9) or executioner caspases (caspase-3, -6, and -7).^99^

During the last 2 decades, some seaweed phlorotannins have been reported to trigger apoptosis. Thus, it has been recently reviewed that seaweed compounds such as dieckol, phloroglucinol, phlorofucofuroeckol A, and dioxinodehydroeckol promote apoptosis by activating caspases.^3^ Although the review will focus on the most recent findings, the first reports from 2009 indicated that dioxinodehydroeckol from brown seaweed *E cava* increased the expression of caspases and pro-apoptotic proteins p53 and Bax and reduced the expression of the anti-apoptotic protein Bcl-2 in breast cancer cells.^76^ Similarly, dieckol from brown seaweed *E stolonifera* stimulated pro-apoptotic Bcl-2 proteins (Bid, Bim, and Bak), which released cytochrome c into the cytosol and activated caspase-3, -6, -7, -8, and -9 in human liver adenocarcinoma HepB3 cells, with no cytotoxic effect in noncancerous cells.^100^ In colon cancer HT-29 cells, the major phlorotannin phloroglucinol induced apoptosis by enhancing the expression of several caspases, Fas, and Bax/Bak, and Bcl-2 proteins with no damaging effects on healthy gut epithelial cells.^101^

A year later, a phlorotannin-rich extract from brown seaweed *E cava* amplified the apoptotic potential of the anticancer drug cisplatin by increasing intracellular ROS and downregulating the anti-apoptotic protein B-cell lymphoma–extra-large (Bcl-xl) in ovarian cancer cells. Interestingly, the phlorotannin extract reduced cisplatin-induced ROS and cell death in normal cells.^102^ The same group reported similar results for dieckol from *E cava* in the same cell line and year.^103^ In human colorectal cancer cells, phlorofucofuroeckol A from brown algae *E bicyclis* provoked apoptosis through upregulation of an apoptosis mediator transcription factor, ATF3.^104^ In a sarcoma 180 (S180) xenograft-bearing animal model, eckol showed pro-apoptosis and anti-proliferation activities that were confirmed by the increased TUNEL (terminal deoxynucleotidyl transferase dUTP nick end labeling)-positive apoptotic cells, the upregulated caspase-3 and -9 expression, and the downregulated expression of Bcl-2 and Bax.^105^

In 2022, Shin and colleagues^65^ reported that a phlorotannin-rich extract from brown algae *E cava* could regulate the production of the pro-apoptotic proteins Bax and caspase-3 in PC-12 cells. The same year, a compound isolated from *E cava*, 6,6′-bieckol, significantly arrested growth in non–small-cell lung cancer cells. The compound also provoked cytotoxicity by enhancing apoptosis via modulation of Bcl-2, Bax, and caspase-3, -8, and -9.^106^ Also, last year, triphlorethol‐A, a phlorotannin isolated from *E cava*, induced a Bax/Bcl‐2 imbalance and activated the caspase cascade and cytochrome c to trigger apoptosis.^107^ Furthermore, some synthetic derivatives from dieckol—6-*O*-acetyl, 6-*O*-benzoyl dieckols, and 6-*O*-alkyl dieckols—showed higher cytotoxicity against an adenocarcinomic human alveolar basal epithelial cell line (A549) vs normal cells,^108^ suggesting that mono-*O* modifications of dieckol could be a potent instrument to improve the anticancer activity of dieckol.

To conclude this chapter, it is worth noting that the regulatory effect of phlorotannins and other seaweed derivatives on apoptosis seems to go both ways since, also last year, a phlorotannin-enriched extract from brown algae *E maxima* mainly containing dieckol and eckmaxol significantly suppressed UVB-induced HaCaT cell death through inhibition of apoptosis provoked by UVB-overproduced intracellular ROS (Table 2).^67^

**Table 2.** Effect of Marine Phenolics on the Prevention of Cancer Focused on Effects Related to Molecular Pathways: Apoptosis, Protein Kinases, and Other Signaling Pathways

Compounds/marine source	Test model	Outcome	Ref
Dioxinodehydroeckol from brown seaweed *Ecklonia cava*	In vitro: MCF-7 human breast cancer cells	-Increased apoptosis -Increased caspases expression and pro-apoptotic proteins p53 and Bax and reduced anti-apoptotic protein Bcl-2 expression -Reduced inflammatory transcription factor NF-κB	^76^
Dieckol from brown seaweed *Ecklonia stolonifera*	In vitro: human liver adenocarcinoma HepB3 cells	-Stimulated pro-apoptotic Bcl-2 proteins (Bid, Bim, and Bak) -Released cytochrome c into cytosol -Activated caspase-3, -6, -7, -8, and -9	^100^
Phloroglucinol	In vitro: colon cancer HT-29 cells	-Induced apoptosis -Enhanced caspase-3 and -8 expression -Altered Bcl-2 protein -Released cytochrome c	^101^
Phlorotannin-rich extract from brown seaweed *E cava* rich in dieckol	In vitro: A2780 and SKOV3 ovarian cancer cell lines In vivo: SKOV3-bearing mouse model	-Improved the efficacy of cisplatin for ovarian cancer by enhancing cancer cell apoptosis via the ROS/Akt/NF-κB pathway -Reduced cisplatin-induced ROS production and cell death in normal cells	^102^
Ethanolic extract from *E.cava* whose main component was dieckol	In vitro: A2780 and SKOV3 ovarian cancer cell lines	-Cytotoxic effects on A2780 and SKOV3 ovarian cancer cells -Induced the apoptosis on SKOV3 cells via Akt and p38 signaling pathways	^103^
Phlorofucofuroeckol A present in brown seaweed *Eisenia bicyclis*	In vitro: LoVo, HT-29, SW480, and HCT116 cells	-Antiproliferative and pro-apoptotic properties -Induced the apoptosis by the upregulation of ATF3	^104^
Eckol	In vivo: sarcoma 180 (S180) xenograft-bearing animal model	-Proapoptotic and antiproliferative activities -Increased TUNEL-positive apoptotic cells -Upregulated caspase-3 and -9 expression -Downregulated Bcl-2 and Bax expression	^105^
Phlorotannin-rich extract from brown algae *Ecklonia cava* and dieckol	In vitro: neuronal pheochromocytoma PC-12 cells	-Increased cell viability -Decreased intracellular oxidative stress -Reduced pro-apoptotic proteins Bax and caspase-3 production -Decreased early and late apoptosis in PC-12 cells	^65^
6,6′-Bieckol from *E cava*	In vitro: non–small cell lung cancer cells	-Induced apoptosis -Modulated Bcl-2, Bax, and caspase-3, -8, and -9	^106^
Triphlorethol-A from *E cava*	In vitro: U251 human glioma cancer cell	-Attenuated cancer cell proliferation -Ameliorated apoptosis thought JAK2/STAT3 and p38 MAPK/ERK signaling pathways	^107^
Synthesized 6-*O*-acetyl and 6-*O*-benzoyl dieckol	In vitro: human alveolar basal epithelial A549 cells vs normal cells	Showed higher cytotoxicity against A549 cells vs normal cells	^108^
*Ecklonia maxima* extract containing dieckol and eckmaxol	In vitro: a) alpha-melanocyte–stimulating hormone-stimulated B16F10 cells; b) UVB-stimulated HaCaT cells; c) UVB-irradiated HDF cells	-Inhibited mushroom tyrosinase and melanogenesis in B16F10 cells -Suppressed UVB-induced HaCaT cell death consistent with inhibition of apoptosis and reduction of ROS -Inhibited collagen degradation and matrix metalloproteinases expression in UVB-irradiated HDF cells	^67^
*Ecklonia cava* ethanolic extracts with dieckol	In vitro: lipopolysaccharide (LPS)-stimulated murine BV2 microglia	-Inhibited LPS-induced NO and PGE2 production as well as iNOS and COX-2 -Reduced NF-κB and DNA-binding in cells and MAPK activation -Suppressed proinflammatory cytokines	^77^
*Ecklonia cava* extract containing dieckol	In vitro: LPS-stimulated murine BV2 microglia	Suppressed receptor activator of NF-κB ligand (RANKL)-induced osteoclastogenesis via MAPK/NF-κB pathway inhibition and HO-1 induction	^82^
Dieckok from *Ecklonia cava*	In vitro: Aβ_25-35_-induced damage in PC12 cells	-Inhibited TNF-α, IL-1β, and PGE2 production at protein level -Downregulated proinflammatory enzymes such as iNOS and COX-2 -Suppressed p38, ERK, and JNK	^113^
Fucofuroeckol-A from brown algae *Eisenia bicyclis*	In vitro: LPS-induced RAW 264.7 mouse macrophages	-Suppressed NO and PGE2 production and iNO and COX-2 expression -Reduced proinflammatory cytokine production (IL-6 and TNF-α and monocyte chemoattractant protein-1) -Reduced NF-κB and MAPL activation.	^86^
An ethanolic extract from brown algae *Sargassum horneri* (Phaeophyceae)	In vitro: LPS-stimulated RAW 264.7 macrophages	-Inhibited NF-κB translocation and binding to DNA -Decreased TNF-α, IL-1, IL-6, iNOS, COX-2, NO, and PGE2	^90^
Dieckol	In vitro: LPS-stimulated RAW 264.7 macrophages	Inhibited PI3/Akt	^115^
Eckol isolated from brown seaweed	In vitro: PM_2.5_-stimulated skin HaCaT cells	-Enhanced ROS generation -Inhibited apoptosis by inhibiting MAPK signaling pathway	^55^
A novel bromophenol (BOS-93)	In vitro: A549 lung cancer cells	-Inhibited PI3K/Akt/mTOR -Regulated MAPK pathway to induce G0/G1 arrest, apoptosis and autophagy	^116^
A new series of bromophenol–thiosemicarbazone hybrids	In vitro: SK-OV-3, Bel-7402 and HepG2 cancer cell lines In vivo: SK-OV-3 cell xenograft model	-Inhibited PARP-1 activity -Anticancer activities -Inhibited tumor growth in SK-OV-3 cell xenograft model	^117^
Bromophenol-thiazolylhydrazone hybrids	In vitro: 4 human cancer cell lines (A549, Caco-2, HepG2, and U87 MG) and 1 normal cell line (HUVEC)	Inhibited the interaction of translation initiation factor eIF4E/eIF4G	^118^
Extract from brown seaweed *Ishige okamurae* (Phaeophyceae) rich in diphlorethohydroxycarmalol and ishophloroglucin A	In vitro: AGE-products, induced oxidative stress in mouse glomerular mesangial cells	-Suppressed ROS production, MGO accumulation, and apoptotic cell death -Modulated proteins expression involved in the receptor for AGEs and Nrf2/ARE signaling pathways	^64^
Eckmaxol isolated from brown algae *Ecklonia maxima*	In vitro: particulate-matter–induced inflammation in MH-S lung macrophages	-Decreased COX-2 and iNOS expression -Downregulated NO, PGE-2, and proinflammatory cytokines (IL-1β, IL-6, and TNF-ɑ) -Suppressed TLR activation -Downregulated signaling of NF-κB	^95^
A novel bromophenol derivative ethyl (E)-4-(2-[2,3-dibromo-4,5-dimethoxybenzylidene]hydrazine-1-carbothioamido)benzoate (DDHCB)	In vitro: breast cancer HCC-1937 cells	Inhibited PARP-1 activity, disrupting its role in DNA repair and genomic stabilization	^124^
bis(2,3,6-tribromo-4,5-dihydroxybenzyl)ether (BTDE)	In vitro: H_2_O_2_-stimulated human lung cancer A549 cells	-Decreased ROS, MDA, and GSSG/GSH ratio and increased SOD activity -Inhibited KEAP1 expression and increased Nrf2 expression and its downstream proteins TrXR1, HO-1, and NQO1 -Reduced migration, invasion, and vasculogenic mimicry in cells	^63^
A phlorotannin-rich extract from brown algae *Ascophyllum nodosum* and *Fucus vesiculosus*	In vitro: human lung A549 cells	-Reduced benzo(a)pyrene-induced CYP1 activity and P2X7 receptor activation -Decreased ROS production	^125^
Eckol	In vitro: glioma stem-like cells	Suppressed stemness and malignancies in glioma stem-like cells	^126^
Phloroglucinol	In vitro: breast cancer MDA-MB231 cells In vivo: mammary fat pads of NOD-SCID gamma mice	-Inhibited mesenchymal phenotypes of basal type breast cancer cells through downregulation of SLUG -Decreased SLUG through inhibition of PI3K/AKT and RAS/RAF-1/ERK signaling -Suppressed the metastatic ability of breast cancer cells to lungs	^127^
Phloroglucinol	In vitro: endothelial progenitor cells (EPCs) In vivo: Lewis lung carcinoma (LLC) tumor–bearing mouse model	-Reduced the migration of endothelial progenitor cells from the bone marrow into peripheral blood -Reduced the number of capillary microvessels in the peritumoral region of in vivo model	^128^
Brown algae *Ecklonia cava*–derived dieckol	In vitro: MCF-7 human breast carcinoma cell	Attenuated MCF-7 human breast carcinoma cell migration	^130^
Dieckol	In vivo: N-nitrosodiethylamine (NDEA)–induced hepatocarcinogenesis in rats	-Reversed hepatic marker enzyme activities -Decreased lipid peroxidative markers -Increased antioxidant cascade -Decreased NDEA concentration in liver.	^131^
Dieckol	In vivo: NDEA-induced hepatocarcinogenesis in rats	Modulated the expression of key molecules that regulate apoptosis, inflammation, invasion, and angiogenesis	^132^
Aqueous extracts from *Ecklonia maxima* (Phaeophyceae) and *Ulva rigida* (Chlorophyta)	In vitro: human liver cancer (HepG2) cells	Showed antiproliferative and apoptotic effect	^133^
Phlorotannins from brown algae *Costaria costata*	In vitro: α-NaGalase produced by duodenal adenocarcinoma and melanoma cells	Inhibited cancer cell–associated immune-suppressive α-NaGalase	^134^

Abbreviations: AGE, advanced glycation end product; COX-2, cyclooxygenase-2; ER, endoplasmic reticulum; ERK, extracellular signal-regulated kinase; GPx, glutathione peroxidase; GSH, reduced glutathione; GSSG, oxidized glutathione; HO-1, heme oxygenase-1; IL, interleukin; iNOS, inducible nitric oxide synthase; JAK2, Janus kinase-2; JNK, c-Jun N-terminal kinases; KEAP1, Kelch-like–ECH-associated protein 1; MAPK, mitogen-activated protein kinases; MDA, malondialdehyde; MGO, methylglyoxal; NF-κB, nuclear transcription factor–kappaB; NO, nitric oxide; Nrf2, nuclear factor–erythroid 2 related factor 2; PARP-1, poly(ADP-ribose) polymerase-1; PGE2, prostaglandin E2; PI3K/Akt, phosphoinositide 3-kinase/protein kinase B; Ref, reference; ROS, reactive oxygen species; SOD, superoxide dismutase; STAT3, signal transducer and activator of transcription 3; TLR, Toll-like receptor; TNF-α, tumor necrosis factor alpha; TUNEL, terminal deoxynucleotidyl transferase dUTP nick end labeling; UVB, ultraviolet B.

### Protein kinases and other signaling pathways

In the last 2 decades, the research on the effect of phenolic bioactive molecules on cell membrane receptors, upstream/downstream-related proteins of signaling pathways, as well as enzymes that regulate cell proliferation, differentiation, apoptosis, and response under both normal and stress conditions has been an emergent issue. Here we present a brief introduction to the main cell signaling pathways that are potentially responsive to the seaweed phenolics.

The signaling pathway of MAPK activates in response to intra- and extracellular signals that trigger the transmembrane tyrosine kinase receptor, resulting in the regulation of target genes. Extensive research has reported 3 MAPK families in mammalian cells: extracellular signal-regulated kinase (ERK), c-Jun N-terminal kinase/stress-activated protein kinase (JNK/SAPK), and p38 kinase.^109–112^ It is very difficult to simplify the regulatory role of these kinases in the cell function, but it is commonly assumed that enhancement of ERK1/2 normally favors cell proliferation, whereas long-term activation of JNK usually results in cell death.

The universal transcription factor cyclic AMP-responsive element-binding protein 1 (CREB) is activated by phosphorylation and facilitates the regulation of many cell processes.^110–113^ PI3K is a family of enzymes implicated in cell growth, proliferation, and survival, as well as differentiation and intracellular trafficking. Triggering of PI3K generates phosphatidylinositol (3,4,5)-trisphosphate (PIP3) and phosphatidylinositol (3,4)-diphosphate.

The PI3K/AKT/mTOR pathway is a crucial intracellular signaling pathway for cell cycle regulation, since it is intimately related to cellular quiescence, proliferation, cancer, and longevity. Activation of PI3K phosphorylates and triggers AKT, localizing it in the plasma membrane,^114^ where activated AKT evokes downstream effects such as activating CREB, localizing FOXO in the cytoplasm, and activating mTOR. Factors that enhance the PI3K/AKT pathway include epidermal growth factor (EGF), insulin-like growth factor (IGF)-1, or insulin.^114^ Although this pathway is essential to promote growth and proliferation over differentiation of adult stem cells, it is found to be hyperactive in many cancers, which results in a reduction in apoptosis and increased cell proliferation.^114^^,^^115^ Poly(ADP-ribose) polymerase-1 (PARP-1), a 113-kDa nuclear protein, is associated with a number of cellular functions, such as DNA repair, transcriptional and post-transcriptional modulation of gene expression, inflammation, and regulation of cell death. Thus, the development of novel PARP-1 specific inhibitors is a promising strategy to achieve effective results in neoplastic processes.^116^ Another family of signaling proteins involved in metabolic regulation is the sirtuins (SIRT), especially SIRT1, which function as histone deacetylases.^110–112^

Pioneering studies on seaweed phlorotannins and bromophenols on signaling pathways reported that treatment of LPS-stimulated BV2 microglial cells with an ethanolic extract of *E cava*, mainly containing dieckol, suppressed the MAPK pathway, an effect that further contributed to the regulation of the biosynthesis of proinflammatory cytokine.^77^ Indeed, brown algae *E cava* dieckol reduced phosphorylation of p38 and ERK, in addition to AKT/IkB-mediated NF-κB inactivation, in different human cell lines.^78–85^ Similarly, *E cava* dieckol showed suppression of p38, ERK, and JNK in a PC12 cell line, a commonly used neuronal-like model system.^113^ Another phlorotannin from brown algae *E bicyclis*, fucofuroeckol A, reduced the LPS-induced MAPK signaling on cultured mouse RAW 264.7 macrophages.^86–89^

Similarly, 6,60-bieckol reduced signaling of JNK/p38 MAPK/Akt pathways in LPS-stimulated RAW 264.7 cells and BV2 microglial culture cells, suggesting that this compound may be a promising therapeutic strategy for the treatment of inflammatory diseases and neoplastic processes in the future.^90^ In the same LPS-stimulated RAW 264.7 macrophage model, dieckol induced inhibition of PI3K/Akt.^115^ One year later, eckol protected human HaCaT keratinocytes from apoptosis induced by particulate matter by activation of MAPK signaling pathway.^55^ Also in 2019, there were interesting data regarding lung cancer from a novel synthetic bromophenol derivative, BOS-93; this compound inhibited PI3K/Akt/mTOR and regulated the MAPK pathway to induce G0/G1 arrest, apoptosis, and autophagy in A549 lung cancer cells.^116^ In addition, in 2019, the same group reported a new series of bromophenol−thiosemicarbazone hybrids with inhibitory PARP-1 activity in addition to multiple anticancer mechanisms to increase their anticancer activities.^117^ The same group also reported in the same year that some bromophenol–thiazolylhydrazone hybrids inhibited the interaction of translation initiation factors eIF4E/eIF4G.^118^ The eukaryotic initiation factor 4E (eIF4E) is a promising drug target for specific anticancer therapy as an approach to overcome drug resistance and promote chemotherapy antitumor efficacy by inhibiting the phosphorylation of eIF4E and eukaryotic initiation factor 4E-binding protein 1 (4E-BP1) and disrupting mitochondrial function through the mTOR/4E-BP1 signaling pathway.^118^

Very recently, methylglyoxal-induced nephrotoxicity was alleviated by an extract from brown algae *I okamurae* (Phaeophyceae) through a reduction in oxidative stress and modulation of the MAPK signaling pathway in mouse glomerular mesangial cells.^64^ Simultaneously, Rajan et al^66^ showed the activation by dieckol of the Nrf2/MAPK signaling pathway in liver cells to reduce liver cancer in animal models.

With regard to the beneficial effects of phlorotannins on glioma/glioblastoma cancer up to 2018, the review developed by Ferreira et al^119^ is strongly recommended. Since then, the phlorotannin triphlorethol‐A isolated from brown algae *E cava* provoked a decrease in human glioma U251 cell proliferation that was mediated by the regulation of p38 MAPK/ERK signaling pathways.^108^

Finally, the most recent data concerning effects of phlorotannins on key signaling pathways indicate that eckmaxol, a phlorotannin isolated from brown algae *E maxima*, showed a protective effect against damage induced by particulate matter in MH-S lung macrophage cells by suppressing the activation of MAPK pathways, including JNK and p38.^95^ Thus, the most recent studies on the subject seem to support the concept of the regulation of signaling pathways by phlorotannins for a new therapeutic strategy for the prevention and treatment of ailments with an inflammatory/neoplastic component (Table 2).^9^

## MOST RECENT ADVANCES ON INHIBITION OF CARCINOGENESIS BY SEAWEED PHLOROTANNINS

Data regarding the inhibition of carcinogenesis, angiogenesis, tumor progression, and metastasis/invasion by seaweed phlorotannins/bromophenols up to 2020 were comprehensively reviewed in 2020^3,4^ and more recently in 2022,^10^^,^^120^^,^^121^ among others. Also, in 2022, Besednova and colleagues^9^ and Rocha and co-authors^122^ revised the inflammatory component in the process with a focus on every molecular mechanism and signaling pathway, whereas Monteiro and coworkers^123^ focused on the eckol family of phlorotannins.

We now present a brief synopsis of the most recent findings regarding genomic instability, angiogenesis, and invasion and metastasis. In 2020, a synthetic bromophenol derivative, ethyl (E)-4–(2-[2,3-dibromo- 4,5-dimethoxybenzylidene]hydrazine-1-carbothioamido)-benzoate (DDHCB), showed excellent inhibitory activity on PARP-1, disrupting its important role in DNA repair and genomic stabilization on breast cancer cells.^124^ With regard to lung cancer, in addition to those results previously described in the section on signaling pathways,^116^^,^^117^ it is worth noting that BTDE reduced migration, invasion, and vasculogenic mimicry in human lung cancer A549 cells.^63^ And in the same year, it was found that a phlorotannin-rich extract from the brown algae *Ascophyllum nodosum* and *F vesiculosus* was able to reduce cytochrome P450 1 (CYP1) activity induced by benzo(a)pyrene, along with the activation of P2X7 receptors. Additionally, the extract considerably decreased the production of ROS in human lung A549 cells.^125^ The antiproliferative and antiangiogenic effects of phlorotannins observed in cell cultures have been mostly confirmed in xenograft models, strongly suggesting their role in suppression of tumor progression in glioma,^126^ ovarian cancer,^103^ and breast cancer^127^ cells. In addition, phloroglucinol reduced the migration of endothelial progenitor cells from the bone marrow into peripheral blood as well as the number of capillary microvessels in the peritumoral region of a lung tumor–bearing mice.^128^ In 2015, Pádua and coworkers^129^ identified phloroglucinol, fucoxanthin, and fucoidan as 3 primary bioactive compounds from brown seaweed that show potential as therapeutic agents against breast cancer. Simultaneously, phloroglucinol was found to attenuate the metastasis of breast cancer cells to the lungs, resulting in a significant increase in the survival time of mice.^127^ Furthermore, in the same year, it was reported that dieckol, derived from brown algae *E cava*, could also reduce the migration of human breast carcinoma cells.^130^ In 2016, Sadeeshkumar and colleagues^131^ demonstrated the protective effects of dieckol against hepatocarcinogenesis in rats. One year later, the same group showed that dieckol can regulate xenobiotic-metabolizing enzymes, cell proliferation, apoptosis, invasion, and angiogenesis during N-nitrosodiethylamine–induced rat hepatocarcinogenesis.^132^ More recently, eckol reduced tumor growth by augmenting pro-apoptotic proteins (caspase-3 and -9) and decreasing anti-apoptotic proteins (Bcl-2 and Bax) in S180 sarcoma tumor–bearing mice.^105^

Since then, few new data have been reported. In research published in 2022,^133^ extracts from *E maxima* (Phaeophyceae) and *Ulva rigida* (Chlorophyta) exerted antiproliferative and cytotoxic effects in cultured HepG2 hepatocarcinoma cells; the anticancer effect of the seaweed extracts may involve impaired mitochondria function, generation of ROS, and induction of apoptosis. In 2023, a report was published in which the authors determined that a high-molecular-weight fraction of phlorethols with a degree of polymerization (DP) of 11–23 phloroglucinols, isolated from the brown algae *Costaria costata*, serves as an effective marine-based natural inhibitor of cancer cell–associated immunosuppressive α-N-acetylgalactosaminidase (α-NaGalase), particularly in duodenal adenocarcinoma and melanoma cells.^134^

It is worth mentioning that phlorotannins and derived phenolics are not the only seaweed bioactive components with anticancer potential; in fact, a review from 2022 stated that, among other biological effects, seaweed proteins and peptides also show an anti-tumoral activity (Table 2).^135^ Likewise, the anticancer potential of marine organisms is not restricted to seaweeds, but many other sea plants have shown this capacity; *Mentha aquatica* (Angiosperm) is a recent example.^136^ Finally, it is worth mentioning that the traditional preparation of seaweed extracts rich in phlorotannins/bromophenols and/or administration of specific algae phenolic compounds may today be greatly improved by the use of nanovectors, which not only bring numerous advantages, such as stability, biocompatibility, and cellular uptake, but also have been shown to overcome some cancer-related resistances.^137^ With regard to the acceptability of seaweed-derived products, early this year, in a meta-analysis evaluating the impact of whole seaweed, either consumed as capsules, integrated into food products, or as part of meals, the findings revealed encouraging evidence for healthy effects of seaweed.^138^

## PERSPECTIVE AND CONCLUSION

Algae have evolved to be highly efficient at resource utilization and have proven to be a viable source of nutritious biomass that could be a solution to many of the current food-production issues. In fact, seaweed inherently has the desired qualities of a sustainable food source because it produces highly digestible proteins, lipids, and carbohydrates and is rich in essential fatty acids, vitamins, phytochemicals, and minerals.^139^

As a food, seaweed is already included in a high proportion of meals consumed in Asia, especially Japan and South Korea, but with a world population estimation of 10 billion people in 2050 it is unlikely that feeding this population will be achieved using only land resources.^140^ Among the food resources available in the oceans, seaweed has been identified as 1 of the 50 future foods that will contribute to transforming the global food system.^140^ Seaweed farming, known as seaweed aquaculture, can cheaply be developed in any suitable coastal region, and the marketplace is seeing huge growth in demand for sustainable, plant-based products.^141^ In addition to their nutritional value, seaweeds offer value to the overall aim of optimal nutrition because of their associated health benefits, one of them as an anticancer agent.

The exploration of marine-derived phenolic compounds, particularly phlorotannins and bromophenols, has unveiled a promising frontier in cancer research.^3^^,^^4^ As cancer continues to pose a significant global health challenge, understanding the biochemical and molecular mechanisms through which these compounds exert their effects offers intriguing prospects for the future.

Oxidative stress, driven by environmental factors and unhealthy lifestyles, plays a pivotal role in cancer initiation and progression.^19^ The ability of marine phenolic compounds to scavenge ROS and enhance antioxidant defense systems is well documented. Recent studies have expanded the knowledge, highlighting the role of Nrf2/HO-1 pathways in defending against oxidative stress (Table 1).

Chronic inflammation is recognized as a critical factor in cancer development. The relationship between proinflammatory cytokines and cancer progression has spurred interest in natural compounds that inhibit or reduce inflammation. Seaweed phlorotannins and bromophenols have demonstrated their anti-inflammatory potential by modulating critical factors like NF-κB and TLR signaling pathways (Table 1).

Recent advances in the study of seaweed phlorotannins have shown their potential in regulating critical molecular pathways and their significance in inhibiting carcinogenesis, angiogenesis, tumor progression, and metastasis. Seaweed compounds, particularly phlorotannins, have demonstrated their ability to stimulate apoptosis through multiple pathways, ultimately leading to controlled cell death. Notably, compounds like dieckol, phlorofucofuroeckol A, and dioxinodehydroeckol have shown their potential in activating caspases and modulating key apoptotic proteins. Moreover, the synthetic derivatives of dieckol offer exciting prospects for enhancing anticancer activity (Table 2).

Phenolic bioactive molecules derived from seaweed have shown a remarkable influence on cell membrane receptors, upstream/downstream proteins in signaling pathways, and enzymes that control various cellular processes. The modulation of MAPK, PI3K/Akt/mTOR, and other signaling pathways by phlorotannins reveals their potential in regulating cell functions, especially in cases of hyperactivity seen in cancer. Synthetic derivatives, like DDHCB, and natural compounds, like phloroglucinol, have exhibited their ability to disrupt these pathways, offering new avenues for cancer treatment (Table 2).

Studies into the effect of seaweed phlorotannins on genomic instability, angiogenesis, and metastasis continue to provide exciting insights. Compounds like eckol and phloroglucinol have demonstrated their ability to suppress metastasis and inhibit tumor progression across various cancer types. Additionally, synthetic derivatives, like BTDE, have shown promising results in reducing migration, invasion, and vasculogenic mimicry in cancer cells. Furthermore, extracts from seaweeds have been found to exert antiproliferative and cytotoxic effects on cancer cells, with potential mechanisms involving impaired mitochondria function and induction of apoptosis. Moreover, the discovery of high-molecular-weight phlorethols as effective inhibitors of cancer cell–associated immunosuppressive enzymes opens up new avenues for potential therapeutic interventions.

The integration of more recent findings within the last 5 years has shed light on the potential of marine-derived phenolic compounds in cancer prevention and as adjuvants for existing cancer therapies. However, future studies should explore the specific molecular mechanisms of marine-derived phenolic compounds, particularly in cancer prevention. It will be crucial to understand the complex interplay between inflammation and oxidative stress and their relation to tumor onset and progression. With regard to the polyphenol types, the bioactivity of phlorotannins was much more explored than that of bromophenols; in particular, eckols and their derivatives have been shown to be promising. For this reason, future research should delve deeper into the precise mechanisms underlying the effects of well-characterized fractions of phlorotannins and/or bromophenols to understand their specific bioactivity and to explore potential synergistic interactions between different seaweed compounds and investigate innovative delivery methods such as nanovectors. In addition, the bioactivity evaluated for marine phenolics is almost exclusively based on the data available from in vitro assays or cellular and animal models, and the development of human studies must support the current understanding. Therefore, it is essential to design rigorous clinical trials to confirm the current knowledge about the bioactivity of marine phenols and to substantiate the efficiency and safety of marine phenolics in cancer prevention and as co-treatment. Considering the importance of the bioavailability and metabolism of marine phenolics on their bioactivity, bioavailability studies in humans are also mandatory since these studies are almost nonexistent.

Overall, the anticancer potential of marine-derived compounds remains an exciting and evolving area that requires further exploration. Addressing these gaps will enhance the understanding of seaweed’s anticancer potential and facilitate its integration into holistic cancer therapies.
